# A Case Report on the Communication between Oncologists and Physiatrists in the Establishment of Functional Prognosis while Undergoing Chimeric Antigen Receptor T-cell Therapy

**DOI:** 10.25122/jml-2019-0077

**Published:** 2020

**Authors:** Ekta Gupta, Jack B. Fu, Eduardo Bruera

**Affiliations:** Department of Palliative, Rehabilitation, and Integrative Medicine, University of Texas MD Anderson Cancer Center, Texas, United States of America

**Keywords:** Communication, oncologists, physiatrists, goals, activities of daily living, medical oncology, Activities of daily living (ADLs), CAR-T-cell-related encephalopathy syndrome (CRES), Chimeric antigen receptor (CAR), Cytokine-release syndrome (CRS)

## Abstract

Chimeric Antigen Receptor (CAR) T-cell therapy can have severe toxicities, which include CAR-T-cell-related encephalopathy syndrome (CRES). The patient may present with altered mental status, encephalopathy, seizures, and cerebral edema. Depending on the severity, the recovery process will require rehabilitation.

We present a case and explain how communication between cancer physiatrists, oncologists, and patients can affect the expectations for functional recovery, and the importance of setting goals for recovery in a medically complex population.

We present a patient who underwent aggressive chimeric antigen receptor T cell therapy, causing encephalopathy and complications. He initially required total assistance for mobility and activities of daily living. Physiatry was consulted to assist with the rehabilitation plan of care and disposition. Initially, the oncologist conveyed to the patient he would be walking in two weeks, which was unrealistically optimistic. The patient's physiatrist intervened and discussed these expectations with him, alleviating his emotional distress. His condition improved with inpatient rehabilitation, and he was able to ambulate short distances with modified independence in four weeks.

The involvement of a cancer physiatrist allows for recognition and treatment of complications related to cancer and aggressive therapies, along with an accurate functional prognosis assessment. With improved communication and patient involvement, the patient underwent a successful rehabilitation.

## Introduction

Cancer rehabilitation provides a valuable opportunity to restore function and improve the quality of life in patients with cancer and its complications. In most situations, physiatrists rely on communication between patients and family members, their oncologist, and therapists to develop a plan of care for the patient. Because of the multifaceted nature of cancer, it is essential for physiatrists and oncologists to discuss the patient's prognosis and determine both the appropriateness of and functional goals for an inpatient rehabilitation stay [1]. Using the information provided by the oncologist, physiatrist, and therapists, the patient also creates goals and expectations for the rehabilitation stay. If not met, these expectations can often lead to distress. We present a case in which a conversation between a patient and oncologist led to functional expectations that were different from those expressed by the physiatrist to the patient.

### Subject

The patient had been diagnosed with stage IV mantle cell lymphoma, for which he underwent chemotherapy and immunotherapy. Four years later, he experienced disease progression and entered a clinical trial in which he received a conditioning regimen of fludarabine and cyclophosphamide followed by chimeric antigen receptor (CAR) T cell therapy. On day four, after CAR T cell therapy, he developed severe CAR-T-cell-related encephalopathy syndrome (CRES) with his altered mental status and led to encephalopathy, seizures, cerebral edema, and obtundation, requiring intubation. The patient received intravenous steroids, siltuximab, intrathecal cytarabine, mannitol, and hypertonic saline and required an extraventricular drain, which was later removed. He continued to have hallucinations, memory deficits, dysgraphia, and dysarthria, which improved after 20 days. He also developed the cytokine-release syndrome (CRS), which slowly improved with anti-thymocyte globulin for three days. He was diagnosed with medication-induced myelosuppression with thrombocytopenia and right-arm deep vein thrombosis. He had elevated creatinine kinase levels consistent with steroid-induced myopathy, and a slow steroid wean began. By day 25, the patient had significant improvement in his CRS and CRES.

Physical therapists, occupational therapists, and speech therapists evaluated the patient during his course due to the devastating complications of CAR-T cell therapy. On day 26, he was able to sit up partially supported for about 35 minutes. He required maximum assistance for transfer from supine to sitting, total assistance with two people to transfer from bed to wheelchair with a sliding board, and total assistance for all other activities of daily living (ADLs). Speech pathology placed on him on a mechanical soft (dysphagia III) diet and nectar-thick liquids.

Physiatry was consulted on day 27. The physiatrist's physical examination showed 2+ lower extremity edema, which was improving; 2/5 proximal and 3/5 distal bilateral upper limb strength; 1/5 hip flexion; 2/5 distal bilateral lower extremity strength; and decreased proximal arm and leg range of motion due to weakness. He had an intact sensation to light touch with no proprioceptive difficulty. His substantial proximal weakness was consistent with steroid-induced myopathy along with profound asthenia due to cancer treatment side effects and prolonged hospital immobility. In addition, the patient expressed significant emotional distress at his loss of physical function and quality of life, with fear of how this would affect his ability to return to work.

His oncologist, in the presence of the physiatrist, on day 28, discussed with the patient that he had made significant progress after the CAR T cell toxicities and expected ongoing improvement. The oncologist also communicated his functional expectations for the patient, which included a prognosis of wheelchair mobility over the next week and ambulation using the rolling walker the following week.

After his conversation with the oncologist, the physiatrist discussed the functional goals with the patient. Based on his prior and current level of function, the current goal was modified independent status for ADLs and mobility at the wheelchair level. The physiatrist discussed that the patient would progress to using a rolling walker, but it would most likely be over the course of 4 to 6 weeks. The patient expressed significant emotional distress at these contradictory statements, as his oncologist had predicted a faster recovery.

To clarify this change in duration, we discussed the different aspects of recovery. The patient required medical stability, from both a cardiac and neurological standpoint. He needed the mental capacity to carry over steps learned daily with therapy in order to progress, as well as increased endurance to tolerate three hours of therapy focused on training for independence. Being able to lean forward, position feet with knees flexed at 90 degrees, push off with hands on the wheelchair, and then bear weight while extending hips and knees as one reaches onto the walker to stand, requires core and distal strength as well as processing and recall skills. The physiatrist also discussed optimizing nutrition and protein intake to rebuild muscle mass, improving lower extremity edema with higher pressure compression stockings to absorb fluid weight that may affect movement, and doing exercises in addition to therapies to increase endurance.

### Evolution

**Figure 1:**
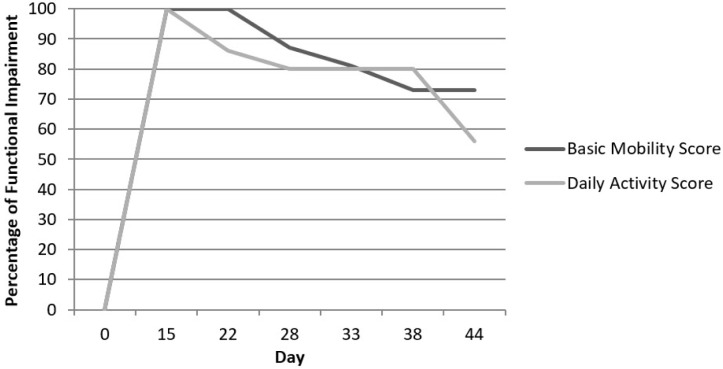
Degree of Functional Impairment: Based off of the Activity Measure for Post-Acute Care (AM-PAC) “6-Clicks” measures of basic mobility and daily activity scores performed during each session by physical and occupational therapists. Using this data, the percentage of functional impairment can then be calculated per day [2].

By day 32, the patient required moderate assistance for sliding board transfers to the wheelchair and was able to go from sitting to supine with moderate assistance. He required total assistance for showers, minimum assistance for upper body dressing, and total assistance for lower body dressing. On day 35, his speech-language pathology was re-evaluated, and he was advanced to a regular diet. On day 40, he required minimum assistance for sliding board to wheelchair transfers and total assistance to go from sitting to standing. As shown in Figure 1, the Activity Measure for Post-Acute Care “6-Clicks” scores were calculated to express the degree of functional impairment [2]. The patient went from fully independent upon admission to entirely dependent, then improved to about 60% to 70% functional impairment over four weeks, still requiring moderate to maximum assistance [3].

He was transferred on day 44 to an outside acute inpatient rehabilitation facility after a discussion with the cancer physiatrist. He was discharged from that facility around day 60, at which point he was ambulating short distances with a rolling walker and performing ADLs with supervision.

## Discussion

Evaluating function is a fundamental component of physical medicine and rehabilitation. Functional expectations take into account a multitude of factors, from current functional status and disease stage and type to treatment complications. It is even more challenging to provide functional prognostic assessments in patients undergoing investigational treatments. While CAR T cell therapy has well documented neurological side effects [4], the return to function and quality of life are more difficult to predict. Cancer physiatrists are beginning to see more profound weakness, asthenia, steroid-induced myopathy, and cognitive and emotional distress in patients who undergo CAR T cell therapy. Early involvement by physiatrists can mitigate some of this distress, and intervention can be provided while medical issues continue to resolve. In this case, it took nearly 16 days after the initial involvement for the physiatrist to deem it appropriate to transfer the patient to an acute inpatient rehabilitation facility for aggressive therapy. During this period, the physiatrist educated the patient and care team on nutrition, core strengthening, and the exercise program required to increase endurance to prosper in inpatient rehabilitation. Once admitted to the acute inpatient rehabilitation, the patient met his goals and ambulated short distances with supervision.

Furthermore, it is essential to realize that a limited understanding of expected functional improvement by the primary team can accidentally increase emotional distress when expectations are not met. In this case, despite the presence of a cancer physiatrist, the primary team provided an excessively optimistic prognosis, causing significant distress for the patient. Well-meaning statements by the oncologist can create unrealistic hopes and expectations and may create a tone of frustration for the rehabilitation stay as well [5]. Studies have shown that physicians who deliver bad news are perceived as less compassionate or worse communicators [6,7], and therefore, it may be better for the physiatrist and primary team to give one unified message to patients. By doing so, we may see improved adherence to the rehabilitation program prescribed by the physiatrist. Inherently, the concern arises that without the presence of a cancer physiatrist, a patient who receives an excessively optimistic prognosis from the oncologist might not receive aggressive rehabilitation. Such a patient might be discharged at an entirely dependent functional level and then potentially have further complications such as falls or blood clots, requiring readmission. The cancer physiatrist plays a valuable role in evaluating, identifying, and preventing such concerns.

Prior studies in patients undergoing rehabilitation for neurological illnesses have shown that physicians tend to focus on the physical (disease) aspects of health-related quality-of-life outcomes, whereas patients tend to focus on the psychological (illness) aspects [8]. This disparity between physician and patient baseline assessments can make setting treatment goals difficult [8]. However, studies have shown that patients with higher levels of involvement in formulating treatment goals maintain their therapeutic gains, and improved communication may affect the process [9]. Unfortunately, the physiatrist may lack the bonding between the patient and the oncologist, whom the patient has been seeing for months if not years. This challenges communication between patients and providers and worsens distress from hearing less optimistic functional prognostic assessments. Earlier involvement of physiatrists may lessen this distress and improve communication; Spill et al. showed that in advanced cancer patients, only 39% of physiatrists compared to 61% of oncologists felt patients adequately understood their prognosis [10]. This is an over twenty percent difference in provider-perceived understanding of medical prognosis, which infers there is likely a difference in interpretation of functional prognosis.

## Conclusion

Novel cancer treatments such as CAR-T cell therapy are becoming more and more prominent as oncologists search for a cure to cancer. These aggressive therapies can have significant functional complications, and while oncologists provide accurate cancer and survival prognoses, they may prefer to consult with a cancer physiatrist so that a unified functional prognosis can be provided. As seen in this case report, a unified functional prognosis will have a beneficial emotional effect on the patient and family and will help the oncologist and physiatrist plan the setting and intensity for the patient's rehabilitation.

## Conflict of Interest

The authors confirm that there are no conflicts of interest.
